# What shapes the trophic niche of European plethodontid salamanders?

**DOI:** 10.1371/journal.pone.0205672

**Published:** 2018-10-18

**Authors:** Enrico Lunghi, Fabio Cianferoni, Filippo Ceccolini, Michael Veith, Raoul Manenti, Giorgio Mancinelli, Claudia Corti, Gentile Francesco Ficetola

**Affiliations:** 1 Department of Biogeography, University of Trier, Trier, Germany; 2 Museo di Storia Naturale dell'Università degli Studi di Firenze, Sezione di Zoologia "La Specola", Firenze, Italy; 3 Natural Oasis, Prato, Italy; 4 CNR-IBAF Consiglio Nazionale delle Ricerche, Istituto di Biologia Agroambientale e Forestale, Monterotondo, Roma, Italy; 5 Department of Environmental Sciences and Policy, University of Milano, Milano, Italy; 6 Department of Biological and Environmental Sciences and Technologies (DiSTeBA), University of Salento, Lecce, Italy; 7 Université Grenoble-Alpes, CNRS, Laboratoire d’Écologie Alpine (LECA), Grenoble, France; Tuscia University of Viterbo, ITALY

## Abstract

The trophic niche is a life trait that identifies the consumer’s position in a local food web. Several factors, such as ontogeny, competitive ability and resource availability contribute in shaping species trophic niches. To date, information on the diet of European *Hydromantes* salamanders are only available for a limited number of species, no dietary studies have involved more than one species of the genus at a time, and there are limited evidences on how multiple factors interact in determining diet variation. In this study we examined the diet of multiple populations of six out of the eight European cave salamanders, providing the first data on the diet for five of them. In addition, we assessed whether these closely related generalist species show similar diet and, for each species, we tested whether season, age class or sex influence the number and the type of prey consumed. Stomach condition (empty/full) and the number of prey consumed were strongly related to seasonality and to the activity level of individuals. Empty stomachs were more frequent in autumn, in individuals far from cave entrance and in juveniles. Diet composition was significantly different among species. *Hydromantes imperialis* and *H*. *supramontis* were the most generalist species; *H*. *flavus* and *H*. *sarrabusensis* fed mostly on Hymenoptera and Coleoptera Staphylinidae, while *H*. *genei* and *H*. *ambrosii* mostly consumed Arachnida and Endopterygota larvae. Furthermore, we detected seasonal shifts of diet in the majority of the species examined. Conversely, within each species, we did not find diet differences between females, males and juveniles. Although being assumed to have very similar dietary habits, here *Hydromantes* species were shown to be characterized by a high divergence in diet composition and in the stomach condition of individuals.

## 1 Introduction

Trophic interactions are key determinants of the structure and dynamics of ecological niches in coexisting species [1–4]. Specifically, the trophic niche defines the role of a species in a local food web, identifying energy transfer routes from food resources [5–7]. The width of the trophic niche is mostly defined by the selectivity of the species [8], which contribute in defining the range of food resources which species are able to feed on [9–11]. The width and other features of a species’ trophic niche are often genetically determined [12,13]. However, species trophic niche is generally characterised by a certain degree of intrinsic plasticity, allowing diet shifts when competition occurs, but also to cope with temporal and local variability of the available resources [8,14]. Indeed, beside the intrinsic characteristics of individuals, the realised trophic niche is strongly related to the ability to persist in an environment where food resources vary in space and time [15], and thus to the capacity to obtain from different subsets of resources the complex combination of elements needed to fulfil physiological and metabolic requirements [16–18]. In wide-ranging species, it is likely that populations forage in environments differing in terms of resource availability and trophic networks [16,19]. Therefore, conspecifics of different populations adapt their feeding habits to the local food availability [5]. In addition, seasonality produces a natural fluctuation of resource, which forces periodic variation in species diet composition [16,20]. A further change in dietary habits occurs throughout individual ontogenesis. Individuals require different sets of nutrients during their life stages, and thus prey selection depends on the nutritional needs [21–24]. Moreover, competition may also play a role in shaping species trophic niche [25–27]. Indeed, when resources are limited, species can switch to a sub-optimal set of resources or, alternatively, change their feeding habits to equally profitable ones to coexist with higher competitors [28–31].

Several studies have been performed on the feeding habits of salamanders, focusing on the diet of the different life stages, habitat, season and differences related to sex and size of individuals [9,11,24,32–34]. However, a study encompassing all the above mentioned aspects has never been performed. Furthermore, in only few cases comparisons have been made between multiple congeneric species living in different areas [31,34]. European cave salamanders (genus *Hydromantes*; subgenus *Speleomantes*) is a group of eight terrestrial salamanders often assumed to be very similar to each other, sharing the majority of their morphological, behavioural and life traits [35–40]. Studies on the diet of European *Hydromantes* are only available for three species [9,41–43]. The present study focuses on the diet of six European species: the five endemic to Sardinia (*Hydromantes flavus*, *H*. *supramontis*, *H*. *imperialis*, *H*. *sarrabusensis*, *H*. *genei*) and one inhabiting mainland Italy (*H*. *ambrosii*) [40]. Considering the potential effects of biological interactions on individual prey selection [29–31], in the present study we focused on allopatric populations, i.e. populations in which closely related species are absent, thus hampering interspecific interactions with them (see Methods). We aimed to produce quantitative and qualitative data on different feeding habits of *Hydromantes* addressing two main questions. *1)* Does the diet differ among *Hydromantes* species? *2)* Do seasonality, sex, and ontogeny affect *Hydromantes* diet? The few data available on the diet of the European *Hydromantes* suggest a generalist trophic niche that nevertheless shows seasonal differences [9,41,43]. Understanding the relationships between ontogeny and seasonality in species diet, a life trait directly related to species survival [44], is of high interest for the conservation of threatened species [45,46], especially in the context of the current climate changes, where seasonal climates can suffer strong alterations [47].

## 2 Materials and methods

### 2.1. Studied species

*Hydromantes* [35] are terrestrial plethodontid salamanders with direct development [40]. Because of their particular physiology [40] they are strongly dependent on environmental conditions [39,48]. When external climate becomes unsuitable (too hot and/or dry), they usually move to underground shelters, where suitable microclimate persists all year round [49]. European cave salamanders have few natural predators and competitors, and are generalists feeding on a wide range of prey captured with their projectile tongue [40,50–53]. All *Hydromantes* have allopatric distribution, except a narrow hybrid zone between *H*. *italicus* and *H*. *ambrosii* [40,54,55] (not considered in this study).

### 2.2. Surveys and data collection

The present study and data collection is authorized by the Italian Ministry of Environment (9384/PNM of 12/05/2015) and Regione Autonoma della Sardegna (n° 6312 of 27/03/2017).Our study originates from three-years fieldwork (September 2015, May/June and September 2016, May/June 2017), and is based on the stomach content of 1,250 salamanders belonging to 19 populations, and on 6,006 recognized prey items (summarized in Table 1) [56]. Populations were sampled during different years and seasons, with surveys covering the periods in which *Hydromantes* activity is the highest [56]. Each population was sampled only once per season to avoid resampling of the same individuals (within each season, all salamanders were sampled the same day) [57]. Resampling of the same individuals during different times (seasons/years) cannot be excluded. During each season, our target was sampling at least 40 salamanders per species, if possible, or the maximum number of available individuals. Overall, the average (± SD) number of sampled individuals from each species was: *H*. *ambrosii* = 64.33 ± 10.02, *H*. *flavus* = 56.25 ± 21.68, *H*. *genei* = 51 ± 17.09, *H*. *imperialis* = 59.25 ± 15.90, *H*. *sarrabusensis* = 42.5 ± 28.77, *H*. *supramontis* = 58.5 ± 11.90). If possible, we tried to obtain a balanced number of individuals from different age class and sex (juveniles, adult males and adult females defined on morphological bases). The present analyses refer only to the consumed invertebrate prey (*N* = 5,996), which represent the main food resource for *Hydromantes* [9,41,43]. Vertebrate items were sporadic (*N* = 10), only represented 0.17% of obtained prey items [56], and were excluded from analyses. Prey items were recognised (when possible) at the order level, with the exception of the Staphylinidae (among Coleoptera), Formicidae (among Hymenoptera) and some different life stages, which were considered separately because of their peculiar ecology along with easy morphological identifications. Complete methodological details and data are available in ref. [58].

**Table 1:** Stomach content of six *Hydromantes* species [58]. For each species: total sampled salamanders; number of empty stomachs; number of not identifiable stomach contents (Not identified); number of recognised prey items.

Species	Sampled salamanders	Empty	Not identified	Prey items
*H*. *ambrosii*	193	38	31	376
*H*. *flavus*	212	23	46	1922
*H*. *genei*	204	71	67	351
*H*. *imperialis*	237	98	108	175
*H*. *sarrabusensis*	170	1	27	3036
*H*. *supramontis*	234	88	91	140

### 2.3. Statistical analyses

All analyses were performed in the R environment [59]. Before data analyses, the linear distance of salamanders from the cave entrance was square-root transformed (hereafter, depth), while the number of prey items was log transformed to improve normality and reduce skewness. We used Generalized Linear Mixed Models (GLMMs; packages lme4, MASS, car, lmerTest [60–63]) to assess the factors determining diet variation. We built GLMMs considering multiple independent variables, to explore the different facets of diet. Dependent variables were: presence/absence of prey (binomial GLMMs; Frequency of empty stomach); average prey size (Prey size), number of consumed prey items (Number of consumed prey) and the Shannon index [64] of stomach contents (Diversity of prey items); all dependent variables were related to data from each sampled salamander. The average size was calculated on 18 prey taxa (Table 2).

**Table 2:** Average size of the consumed prey items (mm ± SE); data based on the size of whole individuals collected by [58].

Group of prey item	*N*	Average length ± SE
Trombidiformes	2	1.54 ± 0.54
Araneae	11	3.34 ± 0.49
Pseudoscorpiones	1	1.43
Julida	2	11.48 ± 3.37
Polydesmida	1	12.06
Isopoda	4	8.18 ± 0.61
Psocoptera	2	1.44 ± 0.44
Hemiptera	1	4.29
Hymenoptera	84	6.94 ± 0.63
Hymenoptera-Formicidae	9	3.87 ± 0.3
Coleoptera	22	3.2 ± 0.36
Coleoptera-Staphylinidae	31	4.89 ± 0.34
Coleoptera-larvae	1	8.34
Trichoptera	1	1.69
Lepidoptera	3	13.38 ± 5.58
Diptera	168	4.27 ± 0.23
Diptera-larvae	5	5.57 ± 1.19
Tricladida	1	7.04

As independent variables, we considered species, season, depth and salamander life history group (adult males, adult females, juveniles). When the life history group resulted significant, we used orthogonal contrast to test whether there are differences between juvenile and adults and, within adults, between males and females; significance between group were assessed through post hoc test. Year and population identity were considered as random factors, to take into account the fact that the same population was sampled multiple times. In GLMMs analyses, we discarded populations in which no individual was detected during one of the two surveyed seasons.

We used non-metric multi-dimensional scaling (NMDS) and the analysis of similarity (ANOSIM with 10,000 permutations; packages MKmisc and vegan [65,66]) to compare diet composition among species and, within each species, between different seasons (autumn, spring) and among sexes/ontogenetic stages (males, females, juveniles). Both NMDS and ANOSIM are non-parametric analyses which evaluate dissimilarity of groups composed by different objects [67]. Here, NMDS plots (with Euclidean distances) where used to visualize differences among groups (species, seasons, or sexes/ontogenetic stages) and put them in relation with descriptor variables. ANOSIM tests where used to verify whether the similarity between groups was higher than that within groups; in particular, for each analyses prey items were used to build a similarity matrix using Bray-Curtis distances. ANOSIM is highly sensitive to the heterogeneity of multivariate dispersion, thus we used the *betadispr* function of the vegan R package to run test whether species show different different levels of multivariate dispersion (999 permutations) for the considered groups. We then used permanova to assess interspecific differences for the composition of diet, as this test is robust to heterogeneity of dispersion [68,69]. To increase the results robustness, when comparing different seasons, we only used populations in which at least three individuals showed identifiable prey items in both seasons.

## 3 Results

### 3.1. Frequency of empty stomachs

**Fig 1:**
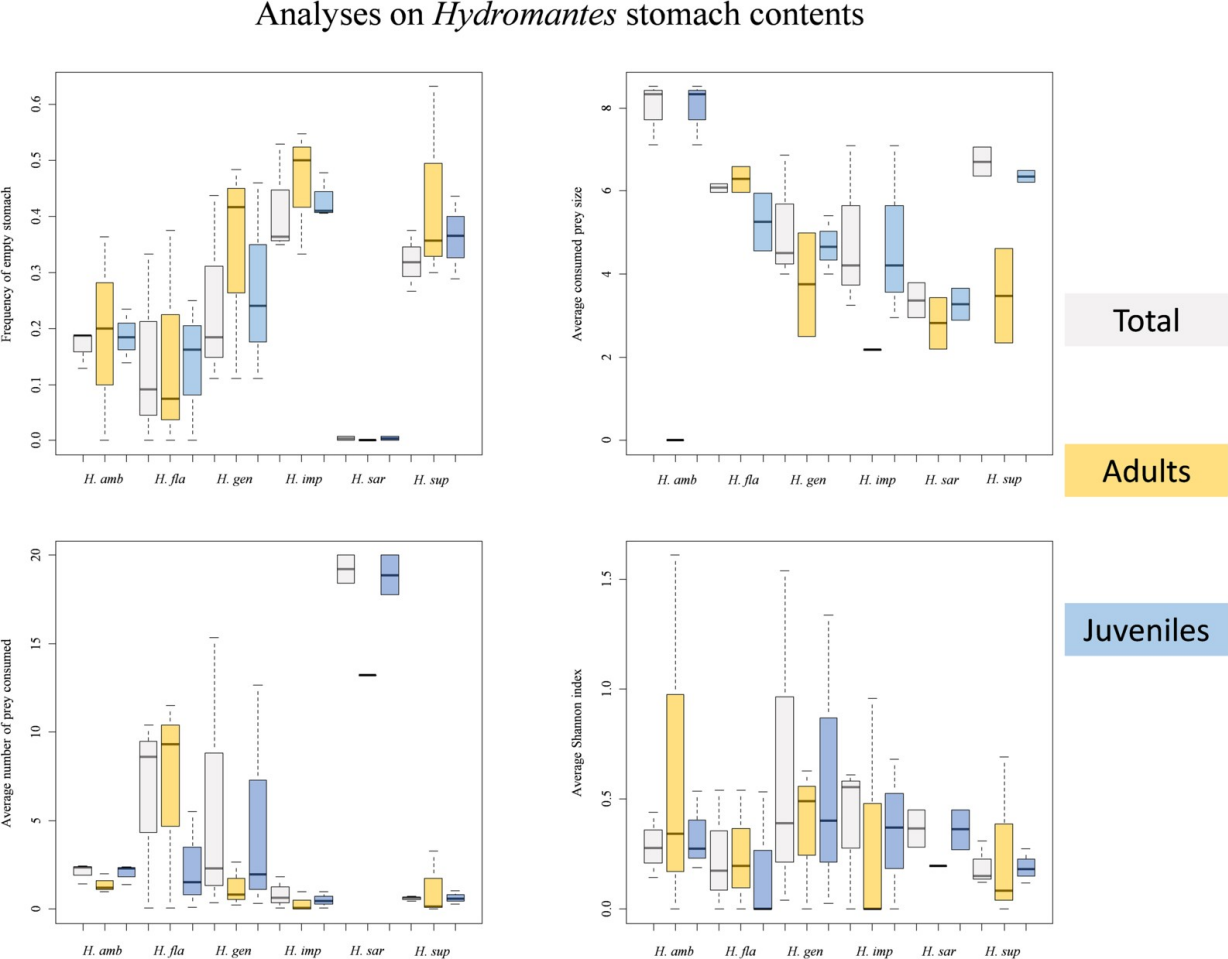
Boxplots showing variation of salamanders’ stomach contents among species and age classes. A) Average frequency of empty stomach; B) average consumed prey size (mm); C) average number of consumed prey; D) average diversity (Shannon index). For each species, averages (±SE) are calculated considering the populations from which salamanders were sampled during each season. Grey = all individuals; orange = adults; blue = juveniles.

The frequency of empty stomachs was significantly lower in spring (*B* = -0.96, *χ*^*2*^ *=* 26.69, df = 1, *P* < 0.001) and in individuals far from the cave entrance (*B* = 0.01, *χ*^*2*^ *=* 9.79, df = 1, *P* = 0.002). We detected significant differences between life history groups (*χ*^*2*^ *=* 11.44, df = 2, *P* = 0.003). Orthogonal contrasts showed a significant higher frequency of empty stomachs in juveniles (*B* = 0.20, *χ*^*2*^ *=* 11.33, df = 1, *P* < 0.001); while we did not detect differences between adult males and females (*B* = 0.06, *χ*^*2*^ *=* 0.41, df = 1, *P* = 0.522). Furthermore, differences between species were also significant (*χ*^*2*^ *=* 64.49, df = 5, *P* < 0.001), as empty stomachs were more frequent in *H*. *supramontis* and *H*. *imperialis*, while they were less frequent in *H*. *flavus* and *H*. *sarrabusensis* (Fig 1A).

### 3.2. Prey size

The size of consumed prey significantly differed between seasons (*B* = 1.69, *F*_*1*, *343*_ = 10.89, *P =* 0.001) and among species (*F*_*5*, *343*_ = 16.78, *P* < 0.001); prey size was the smallest in autumn and in the Sardinian *Hydromantes* species (*H*. *flavus*, *H*. *supramontis*, *H*. *imperialis*, *H*. *sarrabusensis*, *H*. *genei*). Furthermore, we detected significant differences between males, females and juveniles (*F*_*2*, *343*_ = 5.34, *P =* 0.005); juveniles generally consumed prey of smaller size (*B* = -0.59, *F*_*1*, *343*_ = 10.45, *P =* 0.001), while no differences were detected between males and females (*B* = -0.2, *F*_*1*, *343*_ = 0.77, *P =* 0.381) (Fig 1B).

### 3.3. Number of prey

In Table 3 we show the consumed prey categories are reported per salamander species and season. No seasonal difference in the number of consumed prey items was detected (*B* = -0.03, *F*_*1*, *924*.*20*_ = 0.19, *P* = 0.66) (Fig 1C), but the number of consumed prey was higher in individuals close to the cave entrance (*B* = -0.08, *F*_*1*, *174*.*35*_ = 9.94, *P* = 0.002). Furthermore, differences between males, females and juveniles were significant (*F*_*2*, *925*.*82*_ = 3.39, *P* = 0.03); the number of consumed prey was significantly lower in juveniles (*B* = -0.06, *F*_*1*, *907*.*57*_ = 6.14, *P* = 0.013), while no difference between males and females was observed (*B* = -0.03, *F*_*1*, *920*.*16*_ = 0.88, *P* = 0.348). The number of consumed prey significantly varied between species (*F*_*5*, *8*.*14*_ = 10.19, *P* = 0.002). *H*. *sarrabusensis* was the species consuming the highest number of prey items (Fig 1C).

**Table 3:** Qualitative and quantitative analysis of the prey consumed by the six studied *Hydromantes* species. Prey categories and the relative number of recognised prey items per species and per seasons are reported.

Group of prey item	*H*. *ambrosii*		*H*. *flavus*		*H*. *genei*		*H*. *imperialis*		*H*. *sarrabusensis*		*H*. *supramontis*	
	Aut *N* = 152	Spr *N* = 224	Aut *N* = 1407	Spr *N* = 515	Aut *N* = 266	Spr *N* = 85	Aut *N* = 38	Spr *N* = 137	Aut *N* = 1223	Spr *N* = 1813	Aut *N* = 52	Spr *N* = 88
Pulmonata	1	0	0	0	2	0	1	0	0	0	0	0
Sarcoptiformes	6	0	2	0	6	0	0	1	1	0	0	0
Mesostigmata	0	0	1	0	6	0	6	0	0	0	0	0
Trombidiformes	2	0	3	1	3	0	0	0	1	0	0	0
Araneae	22	3	6	2	27	1	3	5	4	6	3	2
Pseudoscorpiones	10	1	2	0	11	0	0	1	1	1	0	0
Opiliones	4	1	0	0	3	0	0	0	0	0	0	0
Lithobiomorpha	1	0	0	0	0	0	0	0	0	0	0	0
Geophilomorpha	1	0	0	0	0	0	0	0	0	0	1	0
Scolopendromorpha	1	0	0	0	0	0	0	0	0	0	0	0
Julida	4	0	4	1	22	0	3	0	0	0	17	2
Polydesmida	0	0	0	1	0	0	0	0	0	0	0	0
Isopoda	17	9	4	0	6	0	0	1	8	1	1	0
Symphypleona	1	0	0	1	8	0	0	0	0	0	0	0
Poduromorpha	0	0	1	0	0	0	0	0	0	0	0	0
Entomobryomorpha	15	1	1	1	1	1	0	0	0	1	0	0
Zygentoma	0	0	0	0	3	0	0	0	0	0	0	0
Ephemeroptera	0	0	0	0	1	0	0	0	0	0	0	0
Odonata_ninfa	0	0	0	0	1	0	0	0	0	0	0	0
Orthoptera	1	0	3	0	6	0	0	0	3	2	0	0
Blattodea	3	0	0	0	1	0	0	0	0	0	0	0
Psocoptera	2	0	1	0	0	1	0	2	7	0	1	0
Hemiptera	1	0	1	0	3	0	0	0	0	1	0	1
Endopterygota_larva	0	0	0	0	0	0	0	0	0	0	1	0
Hymenoptera	4	0	416	250	18	1	6	22	56	18	0	10
Formicidae	9	0	8	11	89	0	14	19	8	6	0	1
Coleoptera	15	6	200	30	16	1	1	24	22	32	4	0
Coleoptera_Staphylinidae	1	1	222	28	4	1	0	6	404	125	3	0
Coleoptera_larva	0	0	1	0	0	0	1	1	0	0	0	0
Neuroptera	0	0	0	0	0	0	0	0	1	0	0	0
Trichoptera_larva	0	0	0	0	1	0	0	0	0	0	1	0
Lepidoptera	0	3	0	1	0	0	0	3	0	3	2	5
Lepidoptera_larva	1	0	0	0	3	0	0	1	1	0	0	0
Diptera	22	187	512	188	25	79	3	51	706	1615	18	63
Diptera_larva	7	11	2	0	0	0	0	0	0	1	0	4
Archaeognatha	0	0	1	0	0	0	0	0	0	0	0	0
Tricladida	0	1	0	0	0	0	0	0	0	0	0	0
Gordea	1	0	1	0	0	0	0	0	0	0	0	0
Nematoda	0	0	10	0	0	0	0	0	0	0	0	0
Haplotaxida	0	0	0	0	0	0	0	0	0	1	0	0

### 3.4. Diversity of prey items

Season significantly influenced diet diversity (*B* = -0.14, *F*_*1*, *407*.*78*_ = 9.22, *P* = 0.002), being higher in autumn; no significant differences were detected for the other variables (species, *F*_*5*, *8*.*69*_ = 0.79, *P* = 0.583; sex/age class, *F*_*2*, *407*.*59*_ = 0.90, *P* = 0.407; depth, *B* = -0.03, *F*_*1*, *122*.*40*_ = 2.28, *P* = 0.134) (Fig 1D).

### 3.5. Prey composition

**Fig 2:**
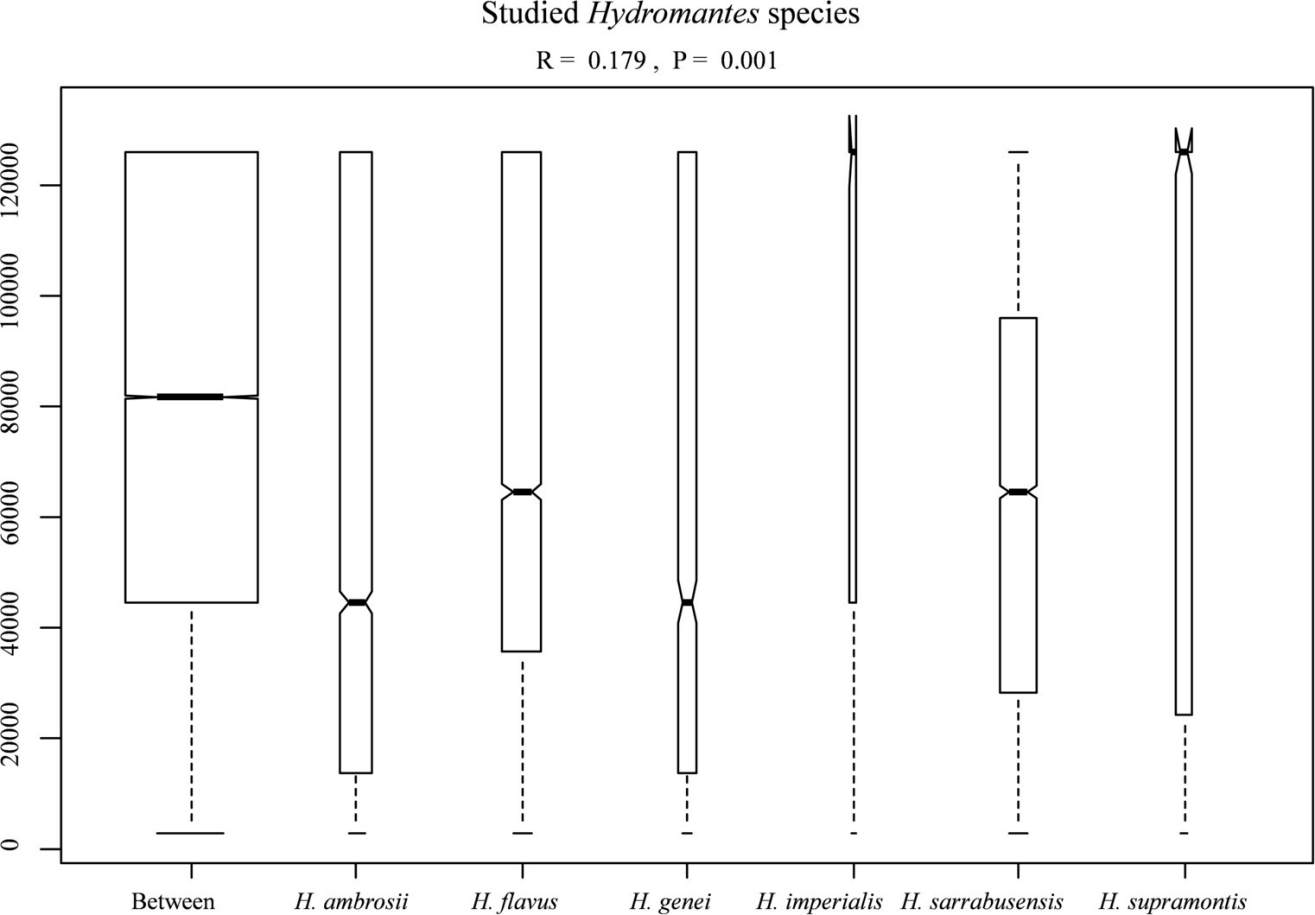
Box whisker plot of ANOSIM analysis comparing the diet of all studied *Hydromantes* species between and within each studied species. Boxes indicates values from 25th (bottom) to 75th (top) percentile; horizontal black line indicate the median; box width is proportional to sample size.

**Fig 3:**
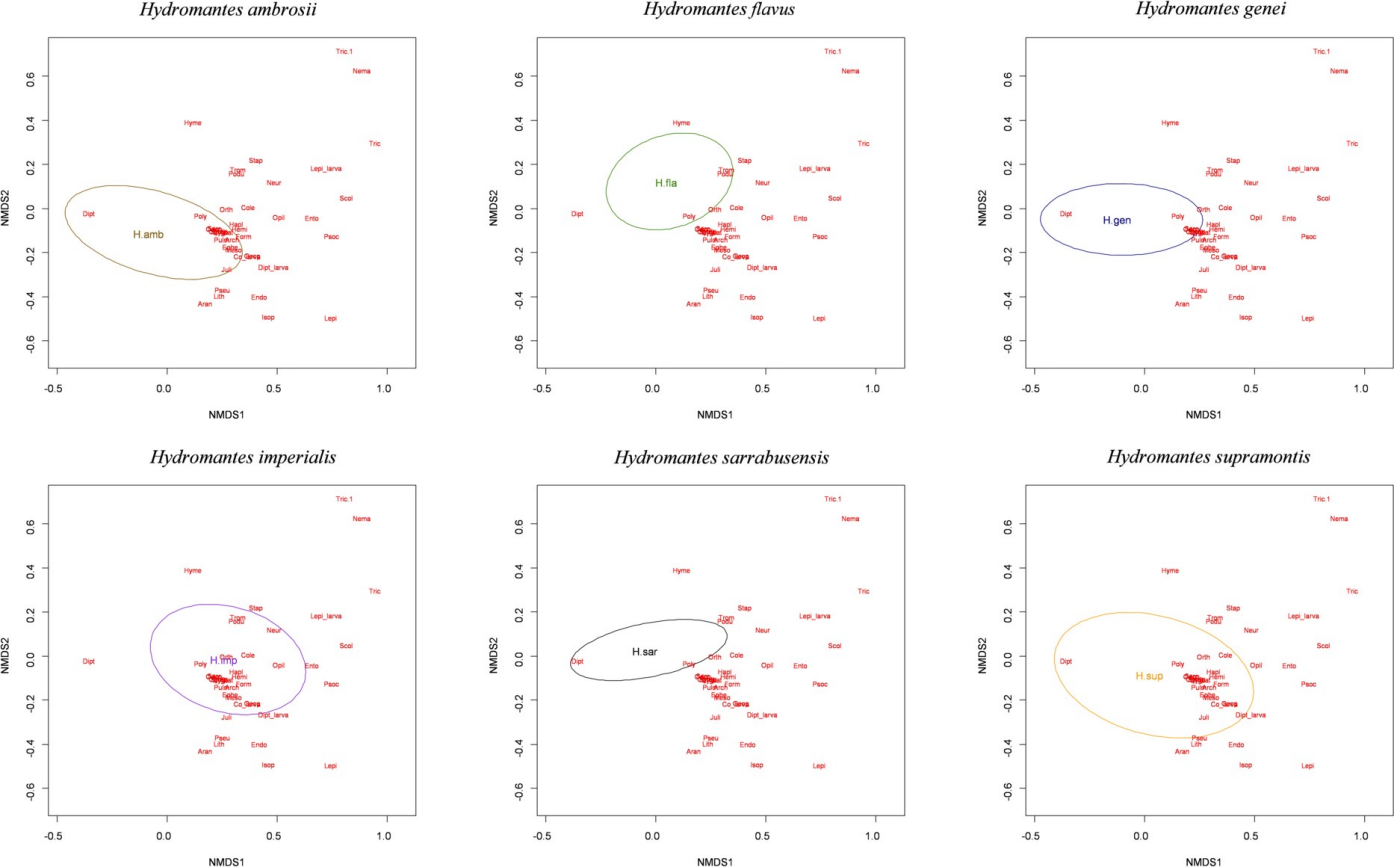
Cumulative NMDS and relative position of each studied species.

The diet of most of the studied species overlapped considerably, with five categories accounting for more than 80% of the diet of all the *Hydromantes* species: Diptera (average ±SE) = 47.8% ± 7.59; Hymenoptera = 11.12% ± 5.17; Hymenoptera Formicidae = 8.13% ± 4.51; Coleoptera = 6.89% ± 2.07; Coleoptera Staphylinidae = 6.32% ± 2.89 (Table 3). The analysis of similarities revealed significant interspecific differences in diet composition (*r* = 0.179, P = 0.001) (Fig 2). The diet of species showed strong heterogeneity of multivariate dispersion (permutation test: *P* = 0.001). In three species (*H*. *genei*, *H*. *supramontis* and *H*. *ambrosii*) within-group variability of diet composition was significantly larger than expected, while variability was significantly lower in *H*. *flavus*, *H*. *sarrabusensis* and *H*. *imperialis* (S1 Fig). Furthermore, permanova confirmed the existence of interspecific differences for the composition of diet (*R*^*2*^ = 0.13, *P* = 0.001). Overall, NMDS identified *H*. *imperialis* and *H*. *supramontis* as the most generalist; *H*. *flavus* and *H*. *sarrabusensis* frequently consumed Hymenoptera and Coleoptera Staphylinidae, while *H*. *genei* and *H*. *ambrosii* mostly Arachnida and larvae of Endopterygota (Fig 3).

**Fig 4:**
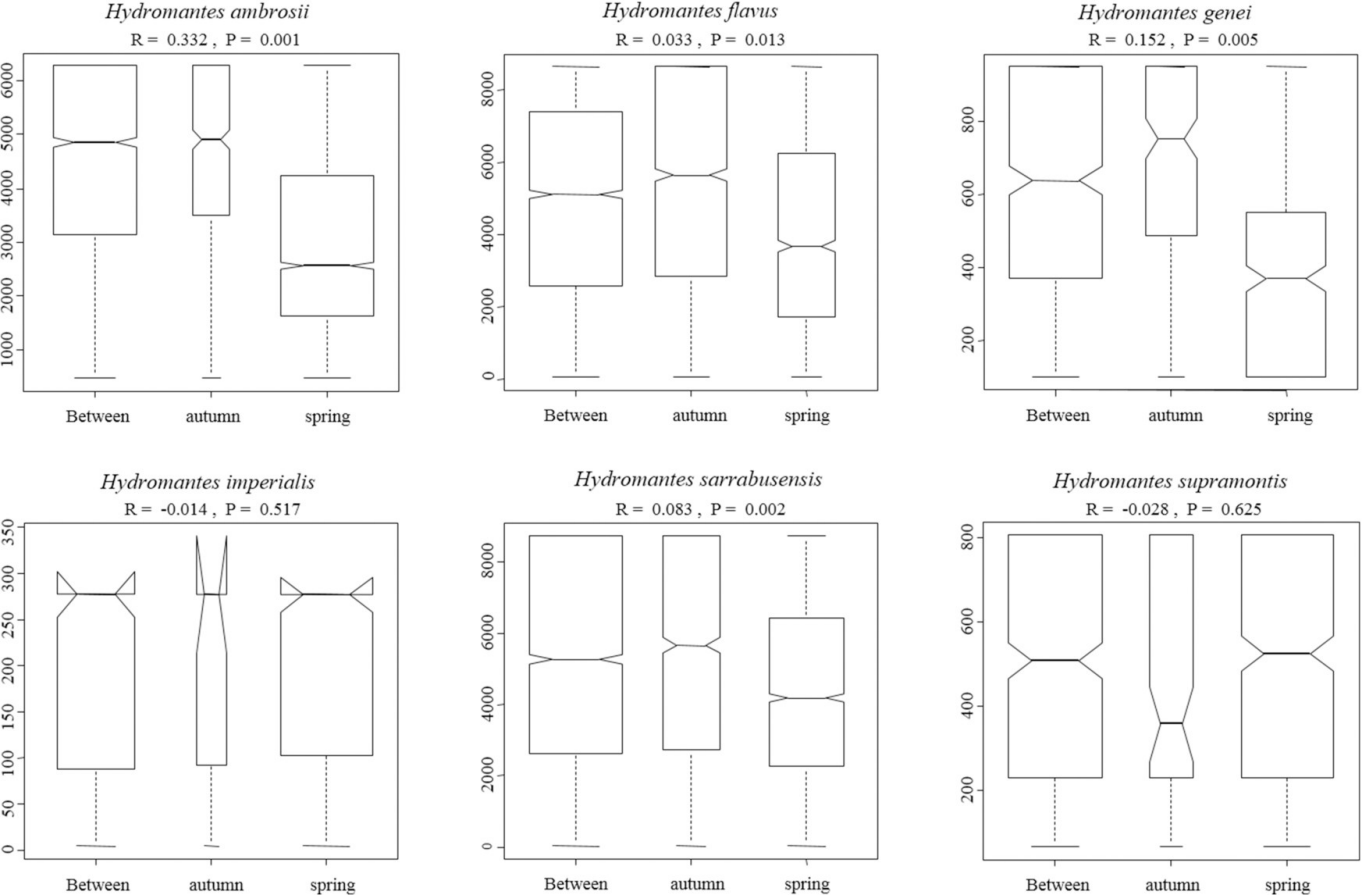
Box whisker plot of ANOSIM analysis comparing the seasonal variation of the diet of *Hydromantes*. Figures show the variation between and within each different seasons (autumn and spring). Boxes indicates values from 25th (bottom) to 75th (top) percentile; horizontal black line indicate the median; box width is proportional to sample size.

ANOSIM analysis highlighted significant diet differences between seasons in four species: *Hydromantes flavus* (*r* = 0.033 and *P* = 0.013); *H*. *supramontis* (*r* = -0.28, *P* = 0.625); *H*. *imperialis* (*r* = -0.014, *P* = 0.517); *H*. *sarrabusensis* (*r* = 0.083, *P* = 0.002); *H*. *genei* (*r* = 0.152, *P* = 0.005); *H*. *ambrosii* (*r* = 0.332, *P* = 0.001) (Fig 4); for three species the hypothesis of an homogeneous distribution of diversity was rejected (*H*. *ambrosii F* = 12.42, df = 1, *P* = 0.001; *H*. *flavus*, *F* = 11.55, df = 1, *P* = 0.01; *H*. *sarrabusensis*, *F* = 4.97, df = 1, *P* = 0.026). In spring, *H*. *flavus*, *H*. *sarrabusensis*, *H*. *genei* and *H*. *ambrosii* mostly consumed Diptera, while in autumn the consumption of Hymenoptera, Coleoptera and ground-welling arthropods, such as Arachnids, Hymenoptera Formicidae, Isopoda and Julida, increased.

**Fig 5:**
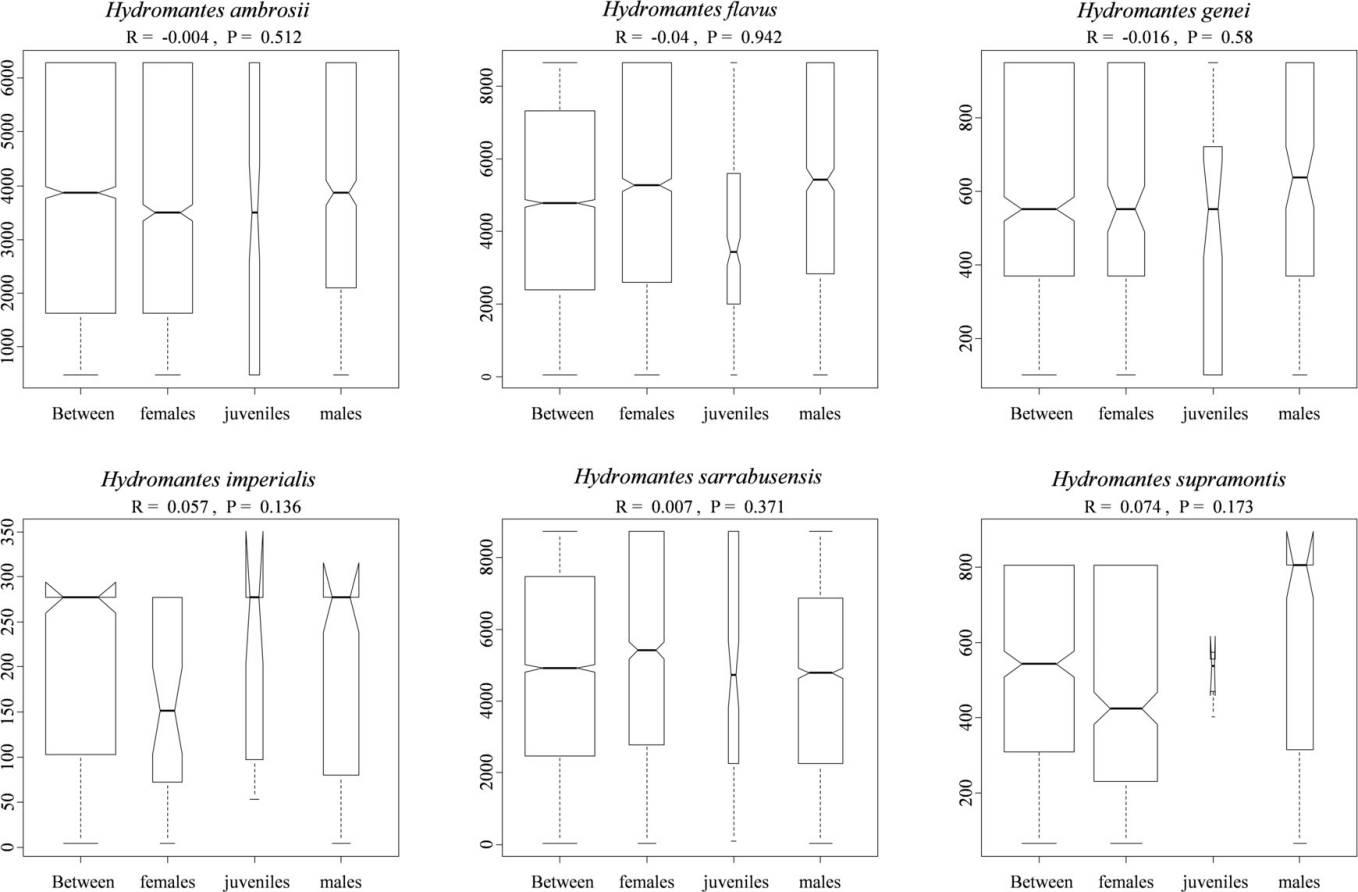
Box whisker plot of ANOSIM analysis comparing the diet of males, females and juveniles per studied species and season. Boxes indicates values from 25th (bottom) to 75th (top) percentile; horizontal black line indicate the median; box width is proportional to sample size.

Finally, no differences in the diet composition were found between males, females and juveniles (all *P* ≥ 0.136; Fig 5).

## 4. Discussion

*Hydromantes* salamanders show a wide trophic spectrum, being able to prey on a very large variety of invertebrate prey (belonging to at least 35 different Orders) (Tab. 3). Our analyses suggest that the feeding ecology of these salamanders is shaped by the interplay between life stage and seasonality, highlighting the complexity of factors determining diet variation.

The frequency of empty stomachs, and the number of prey per salamander, was strongly related with the distance from the cave entrance. Salamanders with empty stomachs and those with few prey items were more frequently found in the deepest sectors of caves [70]. Previous studies on *Hydromantes* salamanders showed spatial segregation, with different age classes and sexes exploiting different cave sectors [71,72]. It has been therefore proposed that microhabitat selection is determined by the trade-off between food availability and microclimate suitability (habitat segregation hypothesis) [72]. Prey availability is usually higher in areas close to the cave entrance [73,74]; on the other hand, microclimatic features are in general more stable and more suitable for salamanders in the deepest cave sectors, where humidity is very high and temperature remains relatively low throughout the year [49,75]. Therefore, individuals with higher energetic demand are expected to select microhabitats close to the cave entrance, where conditions can be sub-optimal but food availability is higher. In such areas predator pressure for this salamanders is generally low, and almost no closely related taxa (i.e., urodela species) are exploiting the same environment, thus limiting the occurrence of intraspecific competition [76,77]. Our findings confirm that the feeding activity of individuals is higher in the areas close to the cave entrance [70], being thus in agreement with the habitat segregation hypothesis [72]

Noticeably, when taking into account the distance from the cave entrance, juveniles showed more frequently empty stomachs, or few small-sized prey items, when compared to adults. This is in agreement with other studies showing that juvenile amphibians are gape limited predators [78]. Moreover, small invertebrates are less detectable and identifiable both because the digestion of small prey is faster and small prey remains might be unrecognizable. Furthermore, only 0.77% of the recognised prey items belongs to taxa without sclerotized parts or shells (Table 3) which seemingly get quickly digested, making identification particularly complicated [9,42]. Salamanders are among the vertebrates with smallest average body size, but small terrestrial salamanders have a key functional role, being one of the major predators of small invertebrates [79,80]. Given that studying the diet of small salamanders is quite complex, alternative approaches, such as genetic identification of prey items, will allow in future studies and advanced resolution of the diet of cave salamander even in absence of identifiable prey items [81].

Generally, Sardinian *Hydromantes* preyed on smaller arthropods compared with *H*. *ambrosii*. This might depend on local availability of prey, or the tendency of some small insects to concentrate in the same place, variables not measured in the present study [82]. Indeed, some small prey like Rove beetles and Diptera tend to gather together in dense groups, thus representing for *Hydromantes* a good opportunity to optimize foraging. Flying prey, captured with the protrusible tongue [83,84], constitutes > 93% of the diet of *Hydromantes*. Some of the detected prey items belong to freshwater habitats, such as nymphs of Odonata and larvae of aquatic Diptera (Table 3). This suggests that *Hydromantes* are also able to prey in shallow water, as recently observed in the Pyrenees in an allochthonous population inhabiting an artificial gallery [85].

Seasonality was one of the major factors determining diet variation in the studied species (Table 3 and Fig 3). Four out of six showed strong seasonal differences in diet composition (between spring and autumn) (Fig 3). In *Hydromantes imperialis* and *H*. *supramontis* (Fig 3D–3F) seasonal differences resulted to be non-significant but this may be related to the limited sample size available (31 and 55 stomach contents only, respectively). At the beginning of the warm season, salamanders increase their foraging activity to face the upcoming aestivation [78]. Considering the high abundance of some prey items in spring/early summer [e.g., *Limonia nubeculosa* Meigen, 1804 Diptera; 73], it is possible that *Hydromantes* focus on the most abundant prey, which may result in a less diverse diet [85,86]. Our study focused on individuals found in caves, where it is assumed that limited seasonality effects occur [49]. However, *Hydromantes* diet strongly varied throughout the year, suggesting that foraging activities mostly occur in cave areas influenced by marked seasonality, and therefore highlighting the importance of climatic conditions outside the cave for species exploiting underground ecosystems [72,74]. Despite being called “cave salamanders”, *Hydromantes* are epigean species that exploit underground habitats to avoid unsuitable outdoor conditions, able to maintain stable population underground [40,49]. However, in underground environments food availability is lower than outdoor [87]. *Hydromantes* foraging activity is therefore likely to occur more frequently in proximity to cave entrance, which is markedly influenced by seasonality [49,70,74]. The methodology adopted in the present work was conceived to avoid individuals resampling during each survey, still it is possible that some individual have been resampled in different periods (seasons/years). However, it is unlikely that this provides a strong bias to our data, given that repeated samples were collected in different periods, that seasonality differences were taken into account into our models, and that population identity was included as a random factor. Furthermore, many of the study populations are large, thus lowering the probability of individuals resampling.

In six stomachs (two males and two females of *H*. *ambrosii*, and two females of *H*. *flavus*), besides the invertebrate prey items, we also detected *Hydromantes* skin rests, probably eaten after moult. *Hydromantes* eggs were also detected in the stomach of two *H*. *imperialis* females (eggs were dissected but no cellular division activity was observed). These eggs could have been removed from the clutch by the mother to avoid any possible contamination of the other eggs with fungi and moulds, as suggested by Lanza et al. [40]. Finally, in a *H*. *ambrosii* female, the remains of one juvenile salamander were also observed. No data on the frequency of true cannibalism are available for *Hydromantes*. Very few cases of possible cannibalism are reported for these salamanders [40]. In one case, Lanza [88] reports the “mysterious disappearance” of some small juveniles placed together with adults in a box in starving conditions; in another one, Voesenek et al. [89] observed in a highly dense population of *H*. *supramontis* a sub-adult vomited by an adult, therefore assuming that high population density may cause cannibalism. *Hydromantes* populations can often reach very high densities. For instance, in some of the studied caves, densities over 10 individuals/m^2^ are frequent [40,75]. We detected only one case of cannibalism in out of 1,250 stomach contents. Therefore, even if true cannibalism exists in *Hydromantes*, it can be considered a very rare phenomenon, also in populations characterized by high population density.

All the six studied species showed remarkable differences in diet composition (Fig 2 and S1 Fig). Multiple factors can determine this variability, such as differences in local food availability, specialization towards different prey items, or additional unexplored environmental factors. Disentangling between these hypotheses is not easy given the allopatric distribution of these species. Accurate estimation of prey availability could be of help to understand the factors involved, but precise measurements of the abundance of invertebrate in underground environments is challenging. Competition with invertebrate predators is an additional, unexplored factor that might affect diet composition. For instance, large spiders are frequent close to the entrance of some caves, and can sometimes reach high abundance. Spiders prey on a wide range of invertebrates and even on juvenile *Hydromantes*, thus potentially affecting patterns of distribution and abundance of available prey [52,73,90]. In future the use of environmental genetics might be of help in assessing cave’s invertebrate diversity [91]. Difference in diet composition between seasons was evident for most of the studies species (Fig 3), however sample size was too small to test whether the pattern of diet shift across seasons was similar among species [56]. The collection of additional data will help to clarify similarity and differences occurring between *Hydromantes* species. Finally, no differences in the diet composition were observed between males, females and juveniles (Fig 4), supporting the hypothesis that a different set of resources is not required during the different life stages [41,43].

## 5. Conclusions

This study provides the broadest assessment of diet variation in European *Hydromantes* species. The diet of these generalist species shows strong seasonal variation, suggesting the important role of the temporal abundance of prey. High turnover of prey likely occur where environmental features are less stable (i.e., shallow cave areas, outdoor), thus highlighting the importance of the connection between the underground and the outdoor environments. Salamanders can have a key functional role in the ecosystems where they live, as they can reach very high abundance and can thus play a key functional role in forest floor communities [80]. For example, salamanders are in a critical intermediate position in the food web, representing a crucial node for the flow of energy and matter between different environments [80,92,93]. Although plethodontids are among the terrestrial salamanders reaching the highest abundances [80,94] most of the studies on the feeding ecology and the functional role of plethodontids are limited to the North American species. Our study is a first attempt to unveil the trophic ecology of different species of European Plethodontids that can be a basis for future investigations highlighting the role of these salamanders in the ecosystems.

## Supporting information

S1 Fig Box whisker plot showing heterogeneity of multivariate dispersion among studied species. (TIF)
