# Supplementary figures and images for: What shapes the trophic niche of European plethodontid salamanders?
Source: PLoS One. 2018 Oct 18;13(10):e0205672. doi: 10.1371/journal.pone.0205672 (PMC6193653; doi:10.1371/journal.pone.0205672)

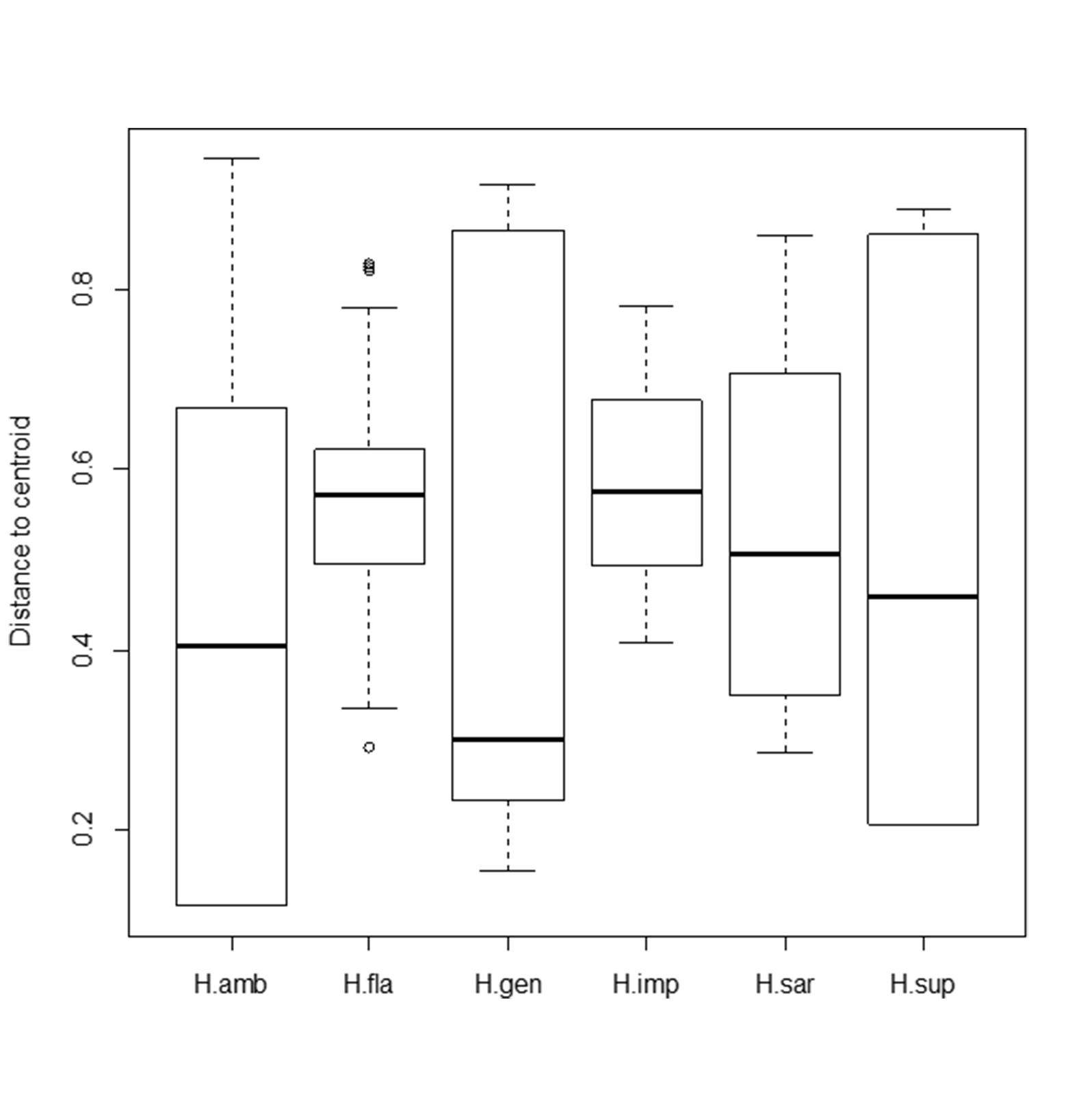

Supplement: S1 Fig — (TIF) [file pone.0205672.s001.tif]
